# Assessment of the effector function of CMV-specific CTLs isolated using MHC-multimers from granulocyte-colony stimulating factor mobilized peripheral blood

**DOI:** 10.1186/s12967-015-0515-z

**Published:** 2015-05-20

**Authors:** Lorea Beloki, Miriam Ciaurriz, Cristina Mansilla, Amaya Zabalza, Estela Perez-Valderrama, Edward R. Samuel, Mark W. Lowdell, Natalia Ramirez, Eduardo Olavarria

**Affiliations:** Oncohematology Research Group, Navarrabiomed - Miguel Servet Foundation, IDISNA (Navarra’s Health Research Institute), Irunlarrea 3, 31008 Pamplona, Spain; Department of Haematology, University College London Medical School, University College London, London, UK; Department of Haematology, Complejo Hospitalario de Navarra, Navarra Health Service, IDISNA (Navarra’s Health Research Institute), Pamplona, Spain

**Keywords:** Allogeneic hematopoietic stem cell transplantation, Cytomegalovirus-specific cytotoxic T cells, Streptamers, Granulocyte-colony stimulating factor, Immunotherapy

## Abstract

**Background:**

Adoptive transfer of CMV-specific T cells has shown promising results in preventing pathological effects caused by opportunistic CMV infection in immunocompromised patients following allogeneic hematopoietic stem cell transplantation. The majority of studies have used steady-state leukapheresis for CMV-reactive product manufacture, a collection obtained prior to or months after G-CSF mobilization, but the procurement of this additional sample is often not available in the unrelated donor setting. If the cellular product for adoptive immunotherapy could be generated from the same G-CSF mobilized collection, the problems associated with the additional harvest could be overcome. Despite the tolerogenic effects associated with G-CSF mobilization, recent studies described that CMV-primed T cells generated from mobilized donors remain functional.

**Methods:**

MHC-multimers are potent tools that allow the rapid production of antigen-specific CTLs. Therefore, in the present study we have assessed the feasibility and efficacy of CMV-specific CTL manufacture from G-CSF mobilized apheresis using MHC-multimers.

**Results:**

CMV-specific CTLs can be efficiently isolated from G-CSF mobilized samples with Streptamers and are able to express activation markers and produce cytokines in response to antigenic stimulation. However, this anti-viral functionality is moderately reduced when compared to non-mobilized products.

**Conclusions:**

The translation of Streptamer technology for the isolation of anti-viral CTLs from G-CSF mobilized PBMCs into clinical practice would widen the number of patients that could benefit from this therapeutic strategy, although our results need to be taken into consideration before the infusion of antigen-specific T cells obtained from G-CSF mobilized samples.

**Electronic supplementary material:**

The online version of this article (doi:10.1186/s12967-015-0515-z) contains supplementary material, which is available to authorized users.

## Background

Allogeneic hematopoietic stem cell transplantation (allo-HSCT) is usually followed by an immunosuppression period during which patients are susceptible to opportunistic infections, including human cytomegalovirus (CMV) as one of the most common viral-infections [1]. Although pharmacological treatment is routinely used prophylactically or pre-emptively to avoid reactivation or primary infection, anti-viral drugs still have some limitations since they are associated with both toxicity and delayed immune reconstitution [2, 3]. It is widely accepted that CMV-specific cytotoxic T lymphocytes are essential to control virus-related complications after allo-HSCT [4]. Therefore the adoptive transfer of antigen-specific T cells after allo-HSCT has been investigated, showing promising results [5].

The majority of studies that generated virus-specific T cells have used non-mobilized peripheral blood mononuclear cells (PBMCs) isolated from an additional leukapheresis collection from the original allo-HSCT donor, different to that of the peripheral blood stem cell (PBSCs) collection performed after granulocyte-colony stimulating factor (G-CSF; Filgrastim) treatment. This additional leukapheresis harvest, apart from increasing the cost of the procedure and the discomfort caused to the donor, can be difficult to obtain in the unrelated donor setting where donor refusal or logistical and scheduling difficulties can prevent collection [6–8]. Manufacture of virus-specific T cells from the original G-CSF mobilized collection could potentially overcome the difficulties associated with procurement of a second apheresis.

However, G-CSF administration has been associated with a tolerogenic T cell phenotype; it promotes regulatory T cells that produce the anti-inflammatory cytokine IL-10, induces a polarization towards T helper cell type 2 (Th2) differentiation and while inhibiting Th cell type 1 (Th1) differentiation [9, 10] and decreasing the expression of genes associated with cytotoxicity, antigen presentation and graft-versus-host disease [11].

Streptamer technology avoids the strict requirements associated with advanced therapy medicinal product (ATMP) manufacture, due to minimal manipulation of direct selection [12] and holds great promise in the unrelated donor setting, widening the clinical application of this fast and simple methodology [13–15]. In this present study we evaluated the feasibility of generating therapeutic CMV-reactive cytotoxic T cells (CTLs) using Streptamers from G-CSF treated donor samples, and were able to show that CMV-specific T cells directly selected from G-CSF mobilized samples are highly functional, although their potential is slightly reduced when compared to non-mobilized CMV-specific CTLs (CMV-CTLs).

## Methods

### Donor population and ethical statement

This descriptive study was approved by the Institutional Review Board at Complejo Hospitalario de Navarra (CHN), and all donors gave written informed consent in accordance with the Declaration of Helsinki prior to enrolment. All samples were obtained from CMV-seropositive healthy donors who carried the HLA-A*02:01 allele. Serological analysis for CMV was obtained from the Microbiology Service of the CHN and HLA-I typing was done in the Immunology Unit of the CHN.

### G-CSF mobilized and non-mobilized donor sample collection and preparation

Filgrastim treated PBMCs (*n = 3*) were obtained from leukapheresis collected from healthy donors who received 10 μg/kg/day of recombinant G-CSF (Sandoz Biopharmaceuticals, Paris, France) every 12 h starting five days before collection. Leukapheresis were performed with a COBE Spectra continuous flow blood cell separator (COBE Spectra apheresis system, Caridian BCT, Lakewood, CO, USA). Cell products anticoagulated with acid citrate dextrose were collected with a 1.1 ml/min flux in a 500 ml container, and an aliquot of 1 ml was taken from the apheresis collection to perform the assays. Non-mobilized PBMCs (*n = 3*) were isolated from buffy coat samples anticoagulated with citrate phosphate dextrose obtained from anonymized healthy donors in the Blood and Tissue Bank of Navarra (BSTN). PBMCs were isolated by Ficoll-Paque density gradient centrifugation (GE Healthcare Bio-Sciences, Uppsala, Sweden), counted in a Neubauer Hemocytometer using 0.4 % trypan blue staining (Sigma-Aldrich, St. Louis, MO) and cells were resuspended in RPMI 1640 Medium (Life Technologies, Paisley, UK) supplemented with 10 % human AB serum (Lonza, Basel, Switzerland). Approximately 30 × 10^6^ freshly isolated PBMCs were cryopreserved for later use as feeders in functional assays. Briefly, PBMCs were mixed with fetal bovine serum (Lonza) containing 20 % dimethylsulphoxide (WakChemie, Steinbach, Germany), at a 1:1 ratio.

### Myeloid precursor removal by plastic adherence

In order to minimize the presence of non-specific cells in the cellular product, the sample was enriched for CTLs by a plastic adherence process [16]. In brief, 2.25 × 10^8^ cells were plated in sterile 225 cm^2^ A/N flasks with CellBIND Surface (Corning, Corning, NY) at 0.5 × 10^6^ cells/ml in X-VIVO 15 Serum-free cell medium w/o supplements (Lonza). Cells were incubated for 1 h at 37 °C and 5 % CO_2_. Non-adherent cells were carefully collected by aspiration to avoid the disruption of adherent myeloid cellular populations and were washed with Dulbecco’s phosphate buffered saline (dPBS; Sigma-Aldrich) before quantification.

### CMV-specific CTL isolation with Streptamer technology

Two different technical approximations were performed in order to obtain a cellular product of anti-CMV therapeutic T cells; in the first one, CMV-specific T cells were isolated and subsequently expanded in order to obtain enough cells to perform functional assays. In the second approach, the PBMC sample was first enriched in CMV-CTLs by antigen stimulation before their isolation was carried out.

#### Isolation of CMV-specific CTLs by multimer staining and subsequent *in vitro* expansion

For 50 × 10^6^ non-adherent PBMCs, 3.75 μg of Streptamer (ST) Magnetic Beads and 5 μg of ST MHC class I (HLA-A*02:01/CMVpp65-NLVPMVATV Streptamer; both from IBA GmbH, Göttigen, Germany) were incubated overnight at 4 °C in the dark to generate the ST-magnetic bead complex. This complex was added to the cell pellet and incubated for 45 min at 4 °C in the dark. ST+ cells were isolated using a Possel_ds selection program on the AutoMACS Pro separator (Miltenyi Biotec, Bergisch Gladbach, Germany). ST was dissociated from the eluted cells with 1 mM d-biotin (IBA GmbH), or left bound to the cell in order to compare the effect of constant binding of the multimer to the TCR.

Following magnetic enrichment, up to 100.000 ST+ CMV-specific CTLs were co-cultured with 8 × 10^6^ γ-irradiated (30Gy) autologous PBMCs that were pre-loaded with 10 μg/ml CMVpp65_495–503_ peptide (NLVPMVATV) overnight (Proimmune, Oxford, UK). The expansion was carried out in round bottom tissue culture tubes (Falcon BD Biosciences) in RPMI 1640 supplemented with 10 % human AB serum, 1 % penicillin/streptomycin (Lonza) and 10 ng/ml of IL-7 and IL-15 (Miltenyi Biotec). Cells were expanded over 21 days, culture medium was changed every 2 or 3 days and cells split when necessary. Viable cell counts were performed every 2–3 days using 0.4 % trypan blue staining.

#### CMV-specific CTL isolation after enrichment during expansion

Before automated CMV-specific CTL selection, the number of specific T cells was increased by stimulating all PBMCs with the CMVpp65_495–503_ peptide. Briefly, up to 30 × 10^6^ PBMCs were cultured in round bottom culture tubes in the presence of 10 μg/ml CMVpp65_495–503_ peptide and IL-7 and IL-15 as previously described, at a concentration of 5 × 10^5^ cells/ml.

After expansion, up to 1 × 10^8^ cells were stained with the ST-magnetic bead complex and ST+ cells were isolated as previously described.

### Characterization of specificity and immunophenotype of CMV-specific cells

To analyze the phenotype and purity of the fresh isolated products and expanded cells, they were stained with the ST-PE complex. Briefly, 0.75 μg of PE-labelled *Strep*-Tactin and 0.5 μg of antigen-specific MHC I-*Strep* (HLA-A*02:01/NLVPMVATV Streptamer) were incubated during 45 min at 4 °C in the dark to form the ST-PE complex. 0.2 μg of this reversible multimer were added to 1 × 10^6^ cells. The incubation was carried out during 45 min at 4 °C in the dark and afterwards cells were stained with, CD8-FITC (BioLegend, San Diego, USA), CD3-PerCP, CD137-APC (Miltenyi Biotec), and CD4-APC-Cy7 (BD Biosciences, San Jose, USA).

During the culture period, specificity and phenotype of expanded cells were analyzed every 7 days by staining with the ST-PE complex and monoclonal antibodies as previously described, with the addition of CD69 PE-Cy7 and CD57 VioBlue (Miltenyi Biotec).

Furthermore, at the beginning and the end of the expansion the memory phenotype of the cells was analyzed by staining with CD45RA-V450 and CCR7-PE-Cy7 (both BD Biosciences) during 15 min at room temperature.

### Analysis of cell-surface expression of activation markers upon antigenic re-stimulation

Expanded CMV-CTLs cells were re-stimulated with either CMVpp65_495–503_-loaded or untouched feeders, used as CMV-stimulator or control feeders respectively, and activation marker expression was analyzed.

Briefly, autologous PBMCs were thawed out to be used as feeders and labelled with 1 μM carboxyfluorescein diacetate succinimidyl ester (CFSE) to discriminate between feeders and responder CMV-CTLs during flow cytometry acquisition and analysis. Subsequently, they were plated at 3 × 10^6^ cells/ml, and loaded with 10 μg/ml CMVpp65_495–503_ peptide to produce CMV-loaded feeders, or left untouched as control feeders and incubated overnight at 37 °C with 5 % CO_2_.

Expanded CMV-CTLs were re-stimulated with either CMV-loaded or control feeders at a 2.5:1 feeder:responder ratio, with 1 × 10^6^ cells/well in 96 well plates. Samples were incubated for 6 h and afterwards 500,000 cells were stained with anti-human CD8-PerCP, CD137-APC, CD4-APC-Cy7, and CD69-PE-Cy-7 monoclonal antibodies.

### Evaluation of cytokine production in response to re-stimulation with the antigen

To assess the effector activity of CMV-CTL by intracellular cytokine staining, expanded cells were co-cultured for 6 h with CMV-loaded or control feeders labelled with CFSE at a 2.5:1 ratio, in the same way as for surface activation marker expression analysis. The incubation was done in the presence of 1 μg/ml anti-CD28 (BD Bioscience) and 1 μg/ml of brefeldin A (Sigma-Aldrich). Following the incubation, 1 × 10^6^ cells were stained with either PE-conjugated anti-human IFN-γ, IL-2, IL-10, TNF-α, or Granzyme B (BD Biosciences) and with CD8-PerCP and CD4-APC-Cy7 monoclonal antibodies, then fixed and permeabilized (Intrastain; DakoCytomation, Ely, UK), according to the manufacturer’s instructions.

### Flow cytometric analysis and isotype controls

All flow cytometry experiments consisted of five to seven color panels, where a minimum of 50.000 CD3+ cells were acquired. CD3+ cells were defined after gating viable lymphocyte population using the Forward Scatter (FSC) versus Side Scatter (SSC) dot-plot, and cell population percentages were derived from the CD3+ cell gate, except for the memory T cell phenotype that is derived from the CD3 + CD8 + ST+ CMV-CTL gate. Data were acquired on a FACSCanto II flow cytometer (BD) and analyzed using FlowJo version 10 (TreeStar Inc., Ashland, OR, USA). Data were summarized using descriptive statistics such as mean ± standard deviations (SD).

For cytokine and activation marker staining controls, mouse antibodies of matching isotype conjugated with PE, APC or APC-Cy7 (BD Biosciences) were used.

## Results

### CMV-CTL isolation from G-CSF mobilized and non-mobilized samples

**Fig. 1:**
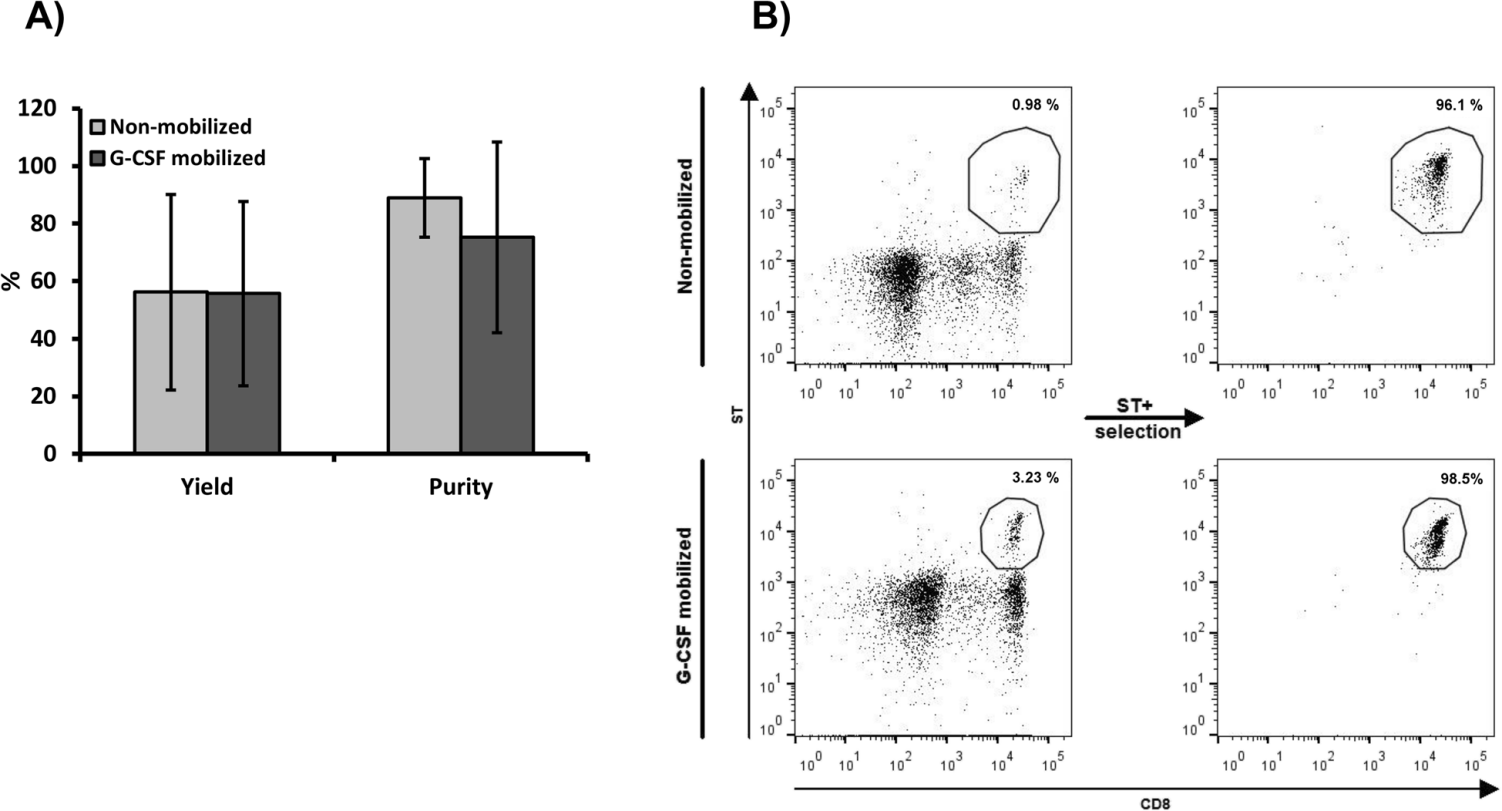
CMV-CTL isolation by MHC-Streptamers. CMV-CTLs, that specifically recognize the CMVpp65_495–503_ peptide of the CMV, were sorted using ST from G-CSF mobilized (*n = 3*) and non-mobilized (*n = 3*) donor samples. (**a**) Mean yield and purity of ST+ cell selection process from original G-CSF mobilized and non-mobilized PBMCs. (**b**) Representative figure of CD3 + CD8 + ST+ cell isolation. ST expression is represented in resting PBMCs and in the positive fraction. Displayed cells were previously gated on CD3+ T cells

Streptamer technology was used to quantify and isolate CMV-CTL from non-mobilized or G-CSF mobilized PBMCs. The mean frequency of CD3 + CD8 + ST+ in the G-CSF mobilized starting material was 1.56 % ± 2.42, compared to 0.5 % ± 0.29 CD3 + CD8 + ST+ in non-mobilized PBMCs. It is noteworthy to mention the high variability between samples, probably due to inter-individual variability because non-mobilized and G-CSF mobilized samples were collected from different individuals. CMV-CTLs were isolated from original samples using Streptamer and the purity of the obtained product was determined as the percentage of ST+ cells in the product, whereas the yield was defined as the absolute number of ST+ cells present in the positive fraction as a proportion of the absolute number of ST+ cells in the sample prior to isolation (Fig. 1a, 1b). In G-CSF mobilized samples, a mean of 20.77 ± 7.83 × 10^6^ PBMCs were used for the isolation, and a cell product made of a mean of 6.5 ± 3.97 × 10^4^ cells with a mean purity of 75.27 % ± 33.15 and yield of 56.28 % ± 26.14 was obtained. From non-mobilized samples, a mean of 37.39 ± 15.73 × 10^6^ PBMCs were used to obtain a cell product made of 7.58 ± 5.82 × 10^4^ CMV-CTL with a mean purity of 88.9 % ± 13.67 and yield of 56.19 % ± 33.95.

### *In vitro* CMV-CTL expansion potential is weakened in G-CSF mobilized samples

**Fig. 2:**
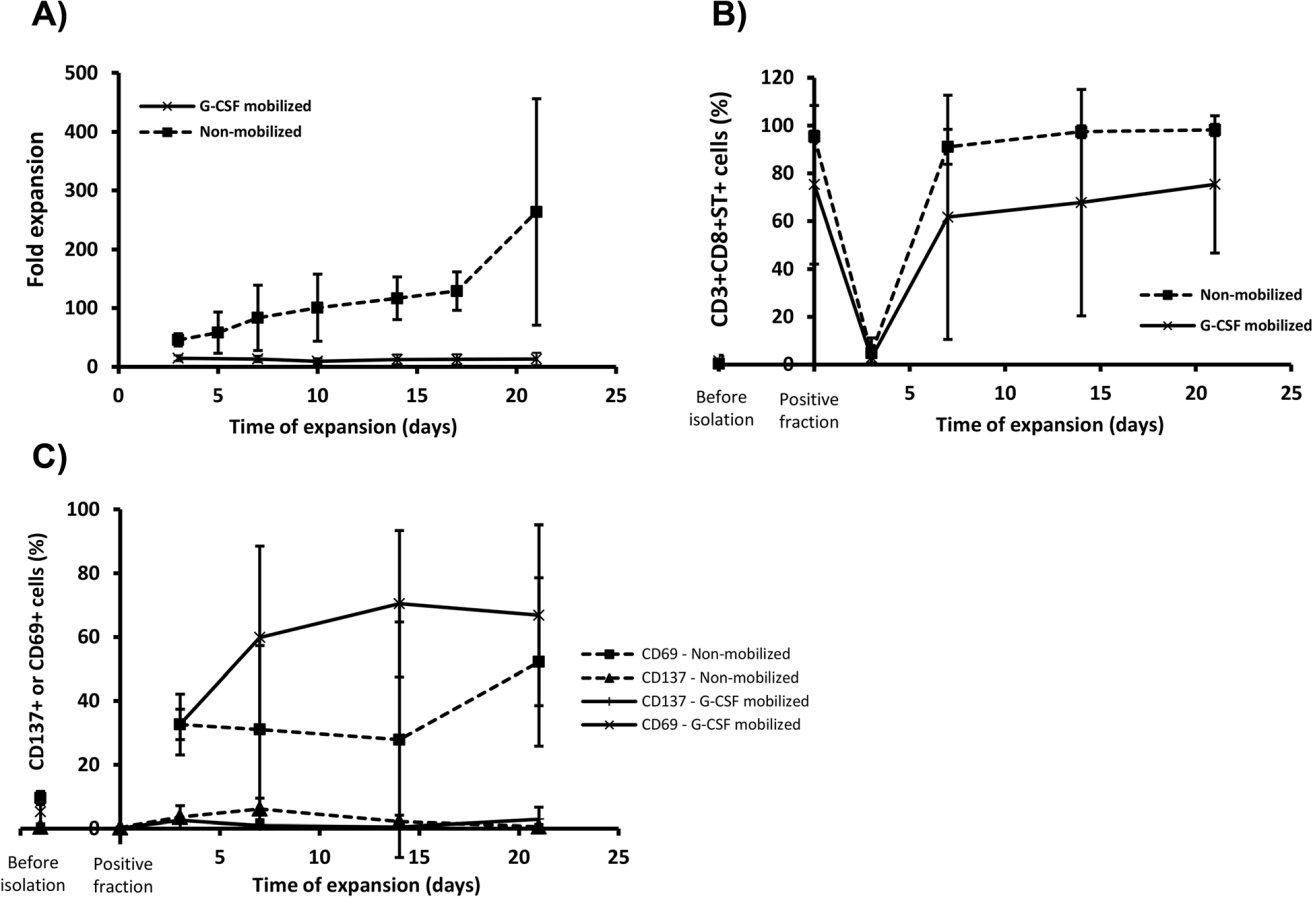
*In vitro* expansion of isolated CMV-CTL and proliferative capacity of expanded cells. CMV-CTL obtained from G-CSF mobilized (*n = 3*) and non-mobilized (*n = 3*) samples were expanded *in vitro* during 21 days co-cultured with irradiated CMV-loaded feeders and homeostatic cytokines. Viable cells were counted every 2–3 days and fold expansion was calculated related to the cells at the beginning of the culture. Represented percentages are obtained from CD3+ T cell gate. (**a**) Mean fold expansion of CMV-CTL obtained from G-CSF mobilized vs non-mobilized PBMCs. Mean percentages of CD3 + CD8 + ST+ cells (**b**) and CD69+ or CD137+ cells (**c**) during culture

To increase the number of isolated CMV-specific T cells in order to perform functional assays, cells dissociated from the streptamer (Additional file 1: Figure S1) were cultured during 21 days in the presence of CMV-loaded feeders and homeostatic IL-7 and IL-15 cytokines. The proliferative ability of cells obtained from G-CSF mobilized samples or from steady state leukapheresis was evaluated (Fig. 2a). The expansion potential of CMV-CTL generated from G-CSF mobilized PBMCs revealed a mean fold expansion of 13.77 ± 10.10, obtaining 1.02 ± 0.36 × 10^6^ CMV-CTLs after expansion. G-CSF mobilized CMV-CTL showed a reduced ability when compared to non-mobilized CMV-CTL (mean fold expansion: 263.75 ± 192.58), where 25 ± 33.69 × 10^6^ CMV-CTL were obtained following expansion.

### CMV-CTLs express the TCR that specifically recognizes the CMVpp65_495–503_ peptide

Cells were phenotypically characterized during expansion by staining with T cell subset markers, Streptamer, and activation markers. Before starting the expansion, the cellular products isolated from G-CSF mobilized PBMCs were predominantly CD3 + CD8+ (77.90 % ± 31.17), and were contaminated with a high percentage of CD3 + CD4+ (16.00 % ± 22.80). Following 21 day culture, expanded cells showed an elevated CD3 + CD8+ T cell population (83.63 % ± 45.08) and a reduced CD3 + CD4+ T cell population (14.49 % ± 19.71). In comparison, streptamer isolated cells obtained from non-mobilized PBMCs, prior to culture were 92.38 % ± 11.88 CD3 + CD8+ and 1.96 % ± 1.21 CD3 + CD4+. Following expansion mean proportion of CD3 + CD8+ and CD3 + CD4+ was 97.67 % ± 1.72 and 1.04 % ± 1.46, respectively. CMVpp65_495–503_-specific TCR expression was analyzed during the expansion (Fig. 2b) and internalization of TCR complex could be observed using streptamer staining after the CMV-loaded feeder addition. The specific TCR was re-expressed from day 7 and expression was maintained until the end of the culture period. After 21 day expansion, the proportion of CD3 + CD8 + ST+ in G-CSF mobilized cells was 75.4 % ± 26.69, and in non-mobilized CMV-CTLs the frequency was 98.17 % ± 2.34.

CD137 and CD69 activation marker expression was analyzed throughout the 21 day culture period (Fig. 2c); CD137 expression was undetectable from the beginning of the expansion and CD69 was heterogeneously and unpredictably changed during the culture, for both G-CSF mobilized and non-mobilized samples.

### G-CSF mobilized CMV-CTL evolve towards a terminally differentiated phenotype during culture

**Fig. 3:**
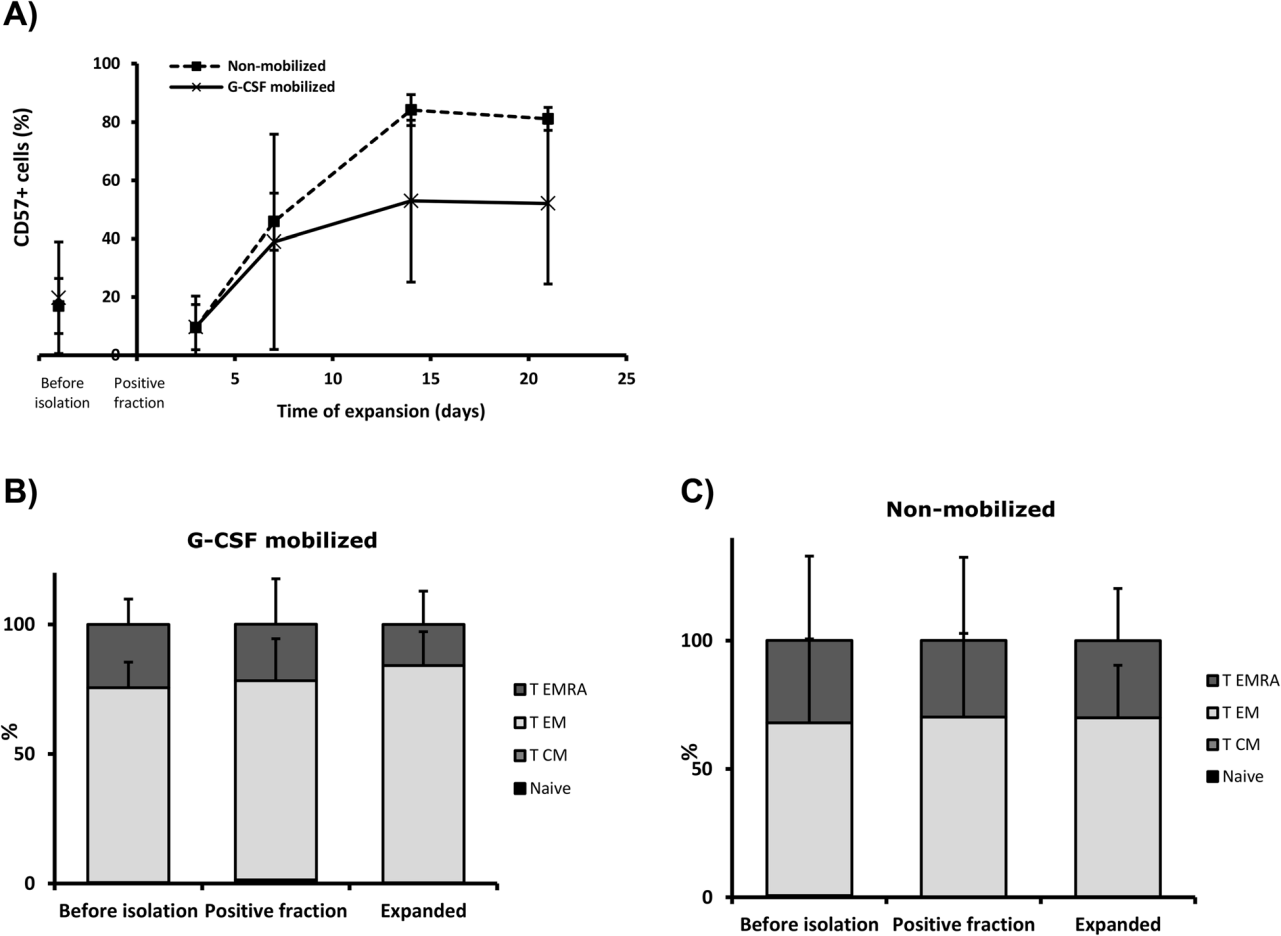
Differentiation status of CMV-CTL in the original sample, after isolation and after expansion. (**a**) Mean CD57 expression of the CD3+ cells was analyzed during *in vitro* culture in G-CSF mobilized (*n = 3*) and non-mobilized (*n = 3*) donor samples. Four subpopulations were distinguished based on their CCR7 and CD45RA memory marker expression: T_N_, T_CM_, T_EM_ and T_EMRA_. Percentage of the four differentiation stages was determined for G-CSF mobilized (**b**) and non-mobilized (**c**) donor samples. They were analyzed in PBMCs before CMV-CTL isolation (*n = 3*), positive fraction of ST+ CMV-CTL isolation (*n = 3*) and CMV-CTL after 21 day expansion (*n = 3*)

Senescence of expanded cells was investigated during the culture period by CD57 expression analysis (Fig. 3a) and showed an increase during the 21 day culture period. At the beginning of the expansion, mean CD57 expression was 9.72 % ± 7.75 and 9.50 % ± 10.85 in G-CSF mobilized and non-mobilized CMV-CTL, respectively. In comparison, after culture mean CD57 expression increased to 52.06 % ± 27.52 in the mobilized CMV-CTL product, and to 81.15 % ± 3.94 in non-mobilized samples.

Memory phenotype of CMV-CTL before isolation, in the positive fraction of ST+ cell products and after expansion was analyzed by staining with CD45RA and CCR7 markers (Fig. 3b, c), and memory subpopulations defined as follows: naïve T cells (T_N_, CD45RA + CCR7+), central memory T cells (T_CM_, CD45RA-CCR7+), effector memory T cells (T_EM_, CD45RA-CCR7-), and terminally differentiated effector memory T cells (T_EMRA_, CD45RA + CCR7-). CMV-CTL obtained from both G-CSF mobilized and non-mobilized PBMCs were predominantly T_EM_ with a considerable T_EMRA_ population (Table 1). Memory phenotype was not notably modified when comparing cells before and after isolation or at the end of the expansion period.

**Table 1:** Differentiation status of directly selected CMV-CTL

	G-CSF mobilized				Non-mobilized			
	T_N_	T_CM_	T_EM_	T_EMRA_	T_N_	T_CM_	T_EM_	T_EMRA_
Before isolation	0.35 % ± 0.07	0.05 % ± 0.07	75.10 % ± 9.90	24.50 % ± 9.76	0.50 % ± 0.87	0.13 % ± 0.23	67.33 % ± 32.66	32.07 % ± 32.98
Positive Fraction	0.50 % ± 0.40	0.93 % ± 1.45	76.77 % ± 16.26	21.87 % ± 17.56	0.00 % ± 0.00	0.03 % ± 0.06	70.27 % ± 32.51	29.73 % ± 32.51
Expanded CMV-CTL	0.30 % ± 0.30	0.00 % ± 0.00	83.8 % ± 13.07	15.90 % ± 12.86	0.13 % ± 0.12	0.07 % ± 0.12	69.77 % ± 20.40	30.03 % ± 20.39

T_N_, T_CM_, T_EM_ and T_EMRA_ subpopulations were determined for G-CSF mobilized and non-mobilized samples in PBMCs before CMV-CTL isolation (*n = 3*), positive fraction of ST+ CMV-CTL isolation (*n = 3*), and CMV-CTL after 21 day *in vitro* expansion (*n = 3*). Percentages were obtained from the CD3 + CD8 + ST+ cell subpopulation

### Expanded CMV-CTL express activation markers and produce pro-inflammatory cytokines and granzyme B

After culture, the effector functional capacity of expanded CMV-CTL was assessed by quantifying activation marker expression and cytokine production upon re-stimulation with the CMVpp65_495–503_ peptide.

**Fig. 4:**
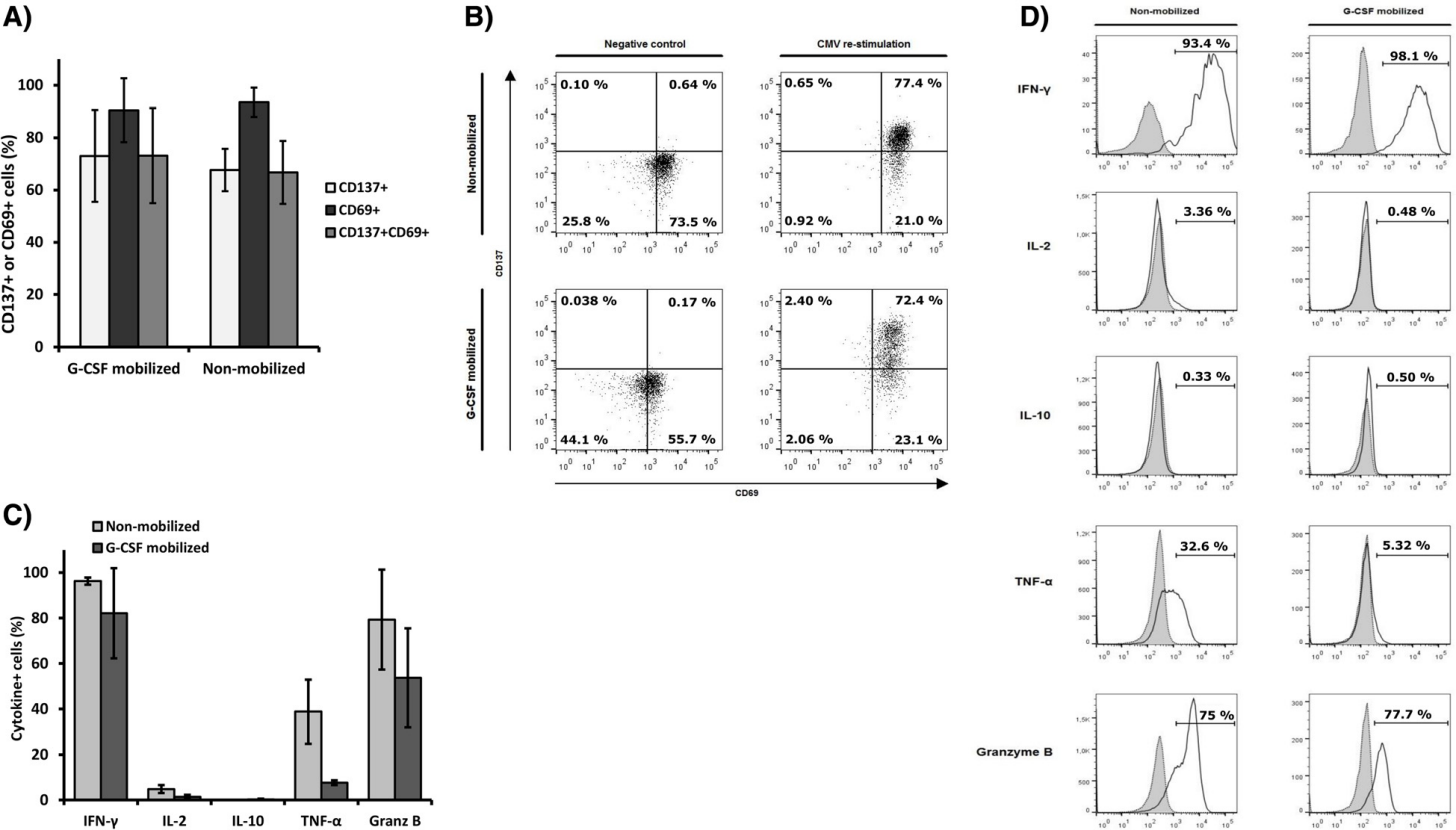
Marker expression and cytokine release after restimulation with antigen. Functionality of expanded CMV-CTL obtained from G-CSF mobilized (*n = 3*) and non-mobilized (*n = 3*) PBMCs was analyzed by CD137 and CD69 expression quantification and cytokine production quantification after re-stimulation with CMVpp65-loaded PBMC (CMV feeders; CMV). (**a**) Mean CD137 and CD69 expression after CMV-loaded feeder re-stimulation, quantified from the CD3+ gate. (**b**) Surface marker expression in a representative re-challenge experiment for both non-mobilized and G-CSF mobilized samples. (**c**) Intracellular cytokine staining (ICS) method was used to determine the percentage of CD3+ cells that produced IFN-γ, IL-2, IL-10, TNF-α, and granzyme B (Granz B, Granzyme B). (**d**) Representative histograms of an ICS experiment for the IFN-γ, IL-2, IL-10, TNF-α, and granzyme B production analysis of G-CSF mobilized and non-mobilized CMV-CTLs, with filled peaks showing matched isotype controls

In G-CSF mobilized CMV-CTL, 73.05 % ± 17.61 cells re-expressed CD137 activation marker after antigenic re-challenge, and 90.50 % ± 12.16 were CD69+. These values were similar to the expression observed in non-mobilized CMV-CTLs (CD137+, 67.67 % ± 8.10; CD69+, 93.53 % ± 5.58) (Fig. 4a, b).

Intracellular cytokine staining following antigenic re-challenge illustrated that CMV-CTL generated from both mobilized and non-mobilized PBMCs were able to secrete pro-inflammatory cytokines and granzyme B (Fig. 4c, d). The secretion cytokine pattern in G-CSF mobilized and non-mobilized CMV-CTLs was similar; G-CSF mobilized CMV-CTLs produced high levels of IFN-γ (82.10 % ± 19.86) and granzyme B (53.67 % ± 21.83). A lower percentage produced TNF-α (7.62 % ± 1.05) and IL-2 (1.44 % ± 0.84), with undetectable levels of IL-10 anti-inflammatory cytokine production. However, the frequency of cytokine secreting cells was slightly reduced when compared to that observed in non-mobilized cells (96.27 % ± 1.57 IFN-γ+, 79.30 % ± 22.00 granzyme B+, 38.83 % ± 14.06 TNF-α+, 4.84 % ± 1.75 IL-2+ and undetectable levels of IL-10).

### Functional CMV-CTL can be manufactured by Streptamer isolation after previous antigen-specific cell enrichment

A different approach for CMV-specific T cell product generation was accordingly assessed, from both mobilized and non-mobilized samples, by first enriching CMV-CTL percentage in the original sample. PBMCs were stimulated with CMVpp65_495–503_ antigen to stimulate CMV-CTL proliferation, expanded in the presence of IL-7 and IL-15, and CMV-CTLs were finally isolated using ST. The function of the obtained cellular product was compared to isolated directly from original PBMCs.

**Fig. 5:**
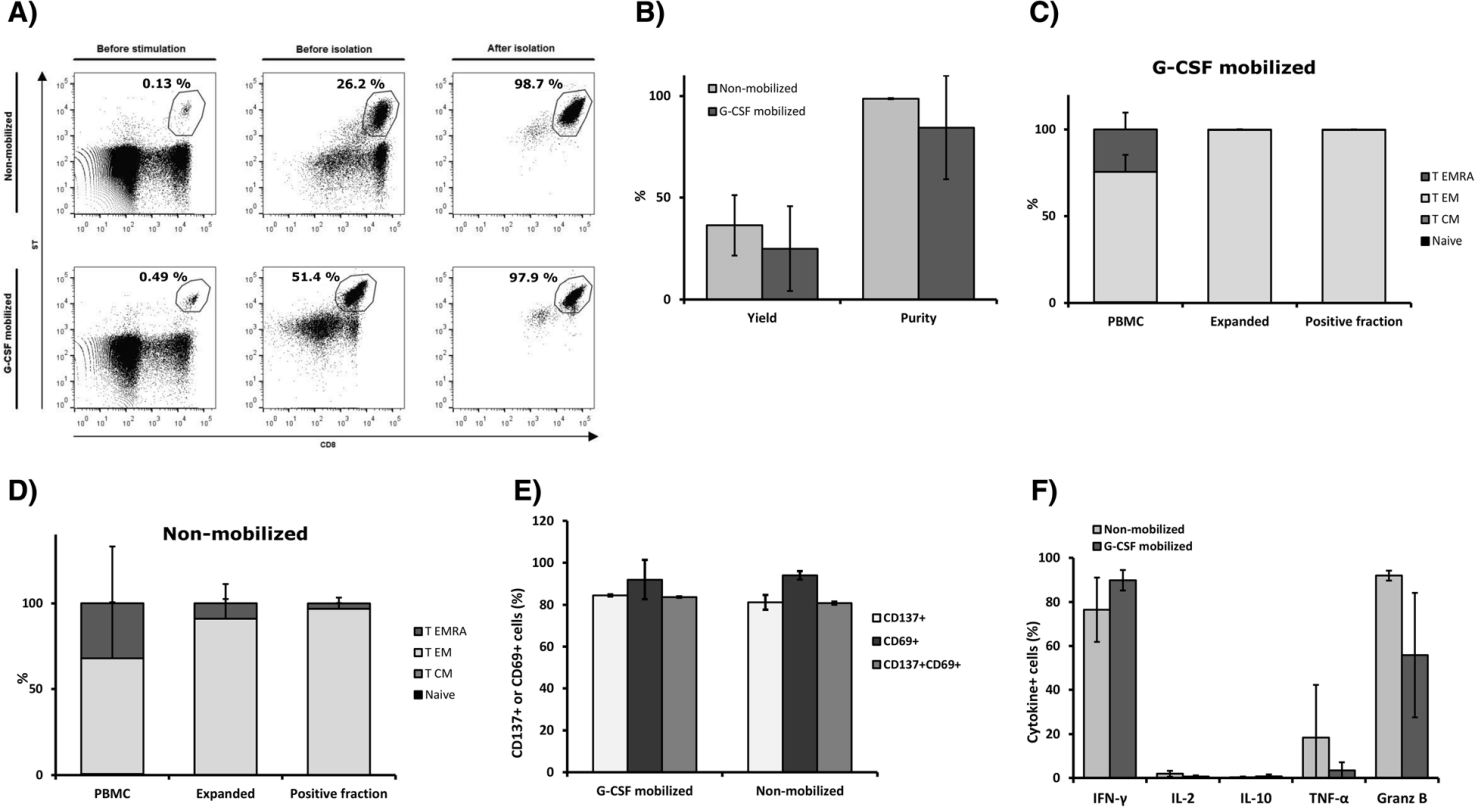
CMV-CTL production by prior stimulation, expansion, and subsequent isolation. CMV-CTL percentage in original PBMCs was increased by peptide stimulation and cells were expanded during 21 days in the presence of IL-15 and IL-7. Afterwards, CMV-CTL were isolated using ST. The process was carried out with non-mobilized (*n = 3*) and G-CSF mobilized (*n = 3*) PBMCs. (**a**) Representative histograms of a CMV-CTL expansion and subsequent isolation. (**b**) Mean yield and purity of ST+ cell selection process for G-CSF mobilized and non-mobilized expanded PBMCs. Memory phenotype of CMV-CTL obtained after isolation, from G-CSF mobilized (**c**) and non-mobilized (**d**) samples. Anti-viral function of isolated cells assessed by activation marker expression (**e**) and cytokine production (**f**) upon peptide re-challenge

Following antigen-specific stimulation of G-CSF mobilized PBMCs, CMV-CTL percentage increased from 1.62 % ± 2.42 at the beginning to 60.23 % ± 48.87 after 21 day culture, with a mean fold increase of CMV-CTL subpopulation of 361.85 (range 23.41 – 791.03). On the other hand, CMV-CTL percentage in non-mobilized PBMCs increased from 0.20 % ± 0.19 to 18.62 % ± 13.35 during culture, and the absolute CMV-CTL number raised by 451.08 fold (range 7.73 – 794.73), similar to G-CSF mobilized CMV-CTLs (Fig. 5a). CMV-specific T cell isolation after expansion of G-CSF mobilized PBMCs showed a mean purity and yield of 84.4 % ± 25.39 and 24.98 % ± 20.80, respectively. CMV-specific T cell isolation from non-mobilized samples showed similar results, with a mean purity of 98.67 % ± 0.25 and yield of 36.39 % ± 14.79 (Fig. 5b). Yield was slightly reduced when compared to direct isolation of CMV-CTL from the original PBMC samples.

In G-CSF mobilized samples, CD57 expression was 19.77 % ± 19.19 in original PBMCs, it was increased to 49.78 % ± 26.58 CD57+ by the end of the expansion, and CVM-CTL isolated after culture were 53.57 % ± 29.75 CD57+. On the other hand, when non-mobilized samples were used, 17.22 % ± 9.90 CD57+ were present in the original samples and the expression was increased to 67.31 % ± 6.07 at the end of the expansion, and after isolation the CMV-CTL product expressed 72.47 % ± 19.49 CD57+. CD57 expression in the CMV-CTL product was higher when compared to CMV-specific cells isolated with ST directly from the original PBMCs, for both G-CSF mobilized (9.72 % ± 7.75 CD57+) and non-mobilized samples (9.50 % ±10.85). However, when directly isolated CMV-CTL were afterwards expanded during 21 days, the differences were lost (G-CSF mobilized CMV-CTL, 52.06 % ± 27.52 CD57+; non-mobilized CMV-CTL, 81.15 % ± 3.94 CD57+). Memory marker expression showed that, in both G-CSF mobilized (Fig. 5c) and non-mobilized (Fig. 5d) donor samples, during culture CMV-CTL T_EMRA_ population decrease, that could denote the poor proliferative capacity and senescence of late-differentiated cells. Therefore, the CMV-CTL product obtained was mainly formed of T_EM_ cells (Table 2).

**Table 2:** Differentiation status CMV-CTL isolated after enrichment by three week expansion in the presence of CMVpp65_495–503_ peptide

	G-CSF mobilized				Non-mobilized			
	T_N_	T_CM_	T_EM_	T_EMRA_	T_N_	T_CM_	T_EM_	T_EMRA_
Before expansion	0.35 % ± 0.07	0.05 % ± 0.07	75.10 % ± 9.90	24.50 % ± 9.76	0.50 % ± 0.87	0.13 % ± 0.23	67.33 % ± 32.66	32.07 % ± 32.98
Expanded PBMCs	0.05 % ± 0.07	0.15 % ± 0.07	99.55 % ± 0.07	0.20 % ± 0.14	0.17 % ± 0.29	0.03 % ± 0.06	90.87 % ± 11.52	8.97 % ± 11.23
Positive fraction	0.00 % ± 0.00	0.10 % ± 0.14	99.70 % ± 0.14	0.15 % ± 0.07	0.00 % ± 0.00	0.10 % ± 0.10	96.70 % ± 3.32	3.20 % ± 3.39

T_N_, T_CM_, T_EM_ and T_EMRA_ subpopulations were determined for G-CSF mobilized and non-mobilized samples in the original PBMC sample (*n = 3*), PBMCs after 21 day *in vitro* expansion (*n = 3*), and positive fraction of CMV-CTL isolation after culture (*n = 3*). Percentages were obtained from the CD3 + CD8 + ST+ cell subpopulation

The functionality of cells isolated after antigen stimulation and culture was similar to the product obtained by direct selection and expansion (Table 3); after re-stimulation with the antigen they re-expressed CD137 and CD69 activation markers (Fig. 5e) and produced high levels of IFN-γ and granzyme B, lower levels of TNF-α and IL-2, and undetectable levels of IL-10 (Fig. 5f). It is important to mention that TNF-α and granzyme B production by CMV-CTL isolated from G-CSF mobilized samples is partly reduced when compared to non-mobilized CMV-CTLs.

**Table 3:** Functional activity of CMV-CTL isolated after three week expansion

	G-CSF mobilized	Non-mobilized
	Activation marker expression	
CD137+	84.50 % ± 0.42	81.20 % ± 3.54
CD69+	92.00 % ± 9.33	94.10 % ± 1.98
CD137 + CD69+	83.70 % ± 0.28	80.80 % ± 0.71
	Cytokine production	
IFN-γ	89.93 % ± 4.65	76.50 % ± 14.64
IL-2	0.61 % ± 0.56	1.91 % ± 1.30
IL-10	0.71 % ± 0.84	0.27 % ± 0.41
TNF-α	3.43 % ± 3.69	18.42 % ± 23.85
Granzyme B	55.83 % ± 28.36	92.07 % ± 2.28

Activation marker re-expression and cytokine production in response to antigenic re-challenge was assessed in CMV-CTLs obtained from G-CSF mobilized (*n = 3*) and non-mobilized (*n = 3*) PBMCs after 21 day expansion in the presence of the peptide. Percentages were obtained from the CD3+ cell gate

## Discussion

Adoptive transfer of CMV-specific T cell products generated from the original PBSC donor has shown promising results in the field of cellular therapy [5, 17]. Most protocols have used non-mobilized apheresis samples as starting material, but the manufacture of the therapeutic product from the same G-CSF mobilized sample used for PBSC collection offers great logistical and regulatory advantages that could avoid the need for successive blood donations and consequently widen the applicability of the adoptive immunotherapy. Recently, the feasibility of CMV-specific T cell generation from a G-CSF mobilized collection has been assessed, confirming the applicability of the procedure [6–8, 18]. Despite these new findings, there is a concern about the effectiveness of the generated cell products due to the immunosuppressive effects associated with G-CSF administration [9, 19, 20]. Furthermore, it has been described that the background staining of MHC-multimers using G-CSF mobilized samples is higher compared to non-mobilized sample staining [20], which would negatively affect the isolation of CMV-CTLs. However, there are currently no studies that evaluate virus-specific T cell production from G-CSF mobilized samples by direct isolation with MHC-multimers. In this study we have investigated whether antigen-specific T cells can be efficiently isolated from G-CSF mobilized samples using reversible Streptamer technology and if T cell products manufactured from mobilized PBMCs with this technology are functionally competent and comparable to those obtained from non-mobilized donors.

After direct CMV-specific T cell isolation from G-CSF mobilized and non-mobilized samples using Streptamers, CMV-CTLs were expanded in culture in order to obtain sufficient cell numbers to perform functional assays. This three week culture could reduce the negative effect of G-CSF or the effect of G-CSF might be abrogated during the expansion, but it was the only option we had to perform functional assessment of isolated CMV-CTLs. CMV-CTLs were dissociated from Streptamers and expanded by co-culture with CMV-loaded autologous feeders and homeostatic cytokines. Feeders were loaded with the CMVpp65_495–503_ peptide since our group has previously described that isolated cells are not able to persist *in vitro* if they are not stimulated with the antigen [unpublished data]. Accordingly, IL-7 and IL-15 were used due to their importance for T cell homeostatic proliferation and survival [21, 22].

Although purity and yield of CMV-CTL isolation from G-CSF mobilized donors was comparable to non-mobilized PBMCs, the proliferative potential of cells obtained from G-CSF mobilized PBMCs appeared to be reduced when compared to non-mobilized samples when selected cells were expanded *in vitro*. A possible explanation could be that the tolerogenic effect induced by G-CSF administration could negatively affect the proliferation of CMV-CTLs *in vitro*. However, when all PBMCs were stimulated with the antigen in order to induce CMV-CTL enrichment before isolation, the expansion potential of CMV-specific cells in G-CSF mobilized samples was comparable to non-mobilized ones. We hypothesize that potential inadequacies in the *in vitro* proliferation protocol resulted in reduced proliferation of G-CSF mobilized CMV-CTLs and that different culture conditions may improve proliferation. Furthermore, in our previous studies where CMV-specific T cells were isolated through CD25, CD137, or CD154 activation marker expression and expanded in the same conditions described here [6, 8, 18], cells isolated from G-CSF mobilized PBMCs were able to proliferate as efficiently as non-mobilized CMV-specific T cells. In these cases, the cellular products were made of CD4+ and CD8+ cell subsets. Therefore, the impaired expansion potential described in this study could denote that G-CSF immunosuppression negatively affects CMV-specific CTL but not helper T cell proliferation, or that isolated antigen-specific CD8+ cells need CD4+ help in order to overcome the immunosuppression exerted by G-CSF, that could be related to the importance of CD4+ T cells in the maintenance of a functional memory CD8+ T cell pool [23]. Nevertheless, CMV-CTLs directly isolated from G-CSF mobilized donor PBMCs may be able to expand more efficiently *in vivo,* since the lymphopenic environment associated with HSCT is likely to be favorable for T cell proliferation [24, 25], and could help in overcoming the problems associated with our proposed *in vitro* expansion protocol.

Differentiation status of CMV-CTLs was also assessed during expansion. CD57 expression was analyzed, a marker of functional immune senescence of T cells that is associated with an impaired proliferation and survival [26, 27]. It could be determined that CD57 expression is increased in both non-mobilized and mobilized samples during expansion, reflecting that CMV-CTL undergo several rounds of cell divisions. Since it has been shown that both proliferative capacity and survival of terminally differentiated CD8 + CD57+ donor T cells are limited following their transfer to HSCT recipients [28], these data suggest that expanded CMV-CTLs cells would potentially persist less in the recipient compared to cells selected and directly infused into the patient. By the end of the expansion, CD57 expression in non-mobilized samples was higher than G-CSF mobilized CMV-CTL, which correlates with the higher fold expansion of non-mobilized CMV-CTLs observed during culture.

The differentiation status of the cells was further characterized by analyzing the immunological phenotype of CMV-CTLs using CD45RA and CCR7 memory markers to distinguish between T_N_, T_CM_, T_EM_, and T_EMRA_ subpopulations [29]. CMV-CTL products, from both G-CSF mobilized and non-mobilized samples were mainly composed of T_EM_ cells and a noteworthy proportion of T_EMRA_ cells, during all the studied stages. These results correlate with the CD57 expression that characterizes T_EMRA_ cells and as well as proportion of T_EM_ cells [30, 31]. These T_EM_ and T_EMRA_ subpopulations which accordingly express CD57+ maintain their proinflammatory cytokine production capacity as well as high cytotoxic effector properties, thereby conferring effective immediate protection [32–34].

After expansion, specificity and effector function of CMV-CTL obtained from both sample sources was analyzed following antigenic re-challenge. The vast majority of cells in the cellular products expressed the TCR recognizing the CMVpp65_495–503_ peptide demonstrating the high specificity of the product. At the beginning of the expansion TCR suffered a downregulation following the activation, which is known to be correlated with antigen sensitivity [35]. A notable proportion of cells expressed CD69 at the end of the expansion, which could be related to the IL-15 present in the medium [36], and upon antigen-specific stimulation a high proportion of CD69+ cells accordingly re-expressed CD137.

Regarding cytokine production upon antigenic re-stimulation, the majority of expanded CMV-CTL from both G-CSF mobilized and non-mobilized samples released the pro-inflammatory cytokine IFN-γ, and lower levels produced TNF-α and IL-2. No IL-10 secretion was observed. This type 1 cytokine secretion profile illustrates the anti-viral functionality of CMV-CTL generated in our protocol [35]. They also produced high levels of the lytic molecule granzyme B, even prior to antigenic re-stimulation, showing their cytotoxic ability since it has been described how the preformation of mature secretory lysosomes containing lytic proteins correlates with the killing ability of cytotoxic cells [37]. This cytokine secretion pattern and granzyme B formation, together with their ability to express activation markers such as CD137 and CD69, illustrates their ability to rapidly activate and induce an effective response after encountering the antigen. Furthermore, it reveals their capacity to confer an immediate effector function, that correlates with the described effector memory T cell phenotype [38].

For the clinical translation of this procedure, we would first eliminate myeloid progenitors of G-CSF mobilized samples performing the plastic adherent process, and subsequently obtained cells would be frozen for storage. When CMV-CTLs were needed, the cells would be thawed and specific cells would be isolated using Streptamer technology, since it has been previously described that CMV-specific T cells can be isolated from frozen cells using MHC-multimers and are totally functional when administered *in vivo* [39].

Finally, we also analyzed an alternative method for the generation of CMV-CTLs. In cases where detectable CMV-CTL in the G-CSF mobilized sample is limited and the purity of the obtained cell product could potentially be reduced [40], CMV-CTL could be enriched in the original sample by antigen-specific stimulation before isolation with the MHC-multimer. The cellular product obtained with this alternative method maintained their functional activity and specificity, although TNF-α and granzyme B production by G-CSF mobilized CMV-CTL was slightly reduced if compared to non-mobilized cells. CD57 expression in CMV-CTL increases during culture, that could potentially impair their long-term maintenance *in vivo* [28]. Furthermore, the cellular product manufactured following antigenic stimulation prior to direct selection would be considered an ATMP with impact on associated regulatory issues and increased costs. Therefore, even though this method could be helpful for the isolation from samples with low antigen-specific cell frequencies, our results do not support it implementation.

## Conclusions

Anti-viral product manufacture from the original G-CSF mobilized PBSC graft offers great advantages mainly in the unrelated donor setting, increasing the number of patients that can benefit from this treatment. This, together with the straightforward CMV-specific T cell production using GMP-grade Streptamers, enhances the broad application into clinical routine of this therapy.

In this study we have been able to manufacture CMV-specific CTLs from G-CSF mobilized donor samples using Streptamer technology that present their anti-viral function, determined by their high specificity and fast and efficient response upon antigen-specific stimulation. However, the capacity to generate pro-inflammatory cytokines and lytic molecules was slightly reduced and their proliferative potential was affected when compared to non-mobilized cell products. Thus, whether the adoptive transfer of a CMV-CTL product from G-CSF mobilized PBMCs can efficiently respond *in vivo* and control CMV disease must be answered in future animal studies and clinical trials.

## Additional file

Additional file 1: Figure S1. Expansion and functionality of CMV-CTL with or without ST dissociation. ST was dissociated (*n = 3*) or left untouched (*n = 3*) from CMV-CTL isolated from non-mobilized samples, and cells were expanded and functionally characterized afterwards. (A) Mean fold expansion of CMV-CTL obtained from non-mobilized samples, with and without ST dissociation using D-biotin. Activation marker expression (B) and cytokine production (C) upon antigenic re-stimulation in expanded CMV-CTL with ST bound to the cell surface or ST dissociated from the TCR.

## Abbreviations

Allo-HSCT: Allogeneic hematopoietic stem cell transplantation
ATMP: Advanced therapy medicinal product
CMV: Cytomegalovirus
CMV-CTL: Cytomegalovirus-specific cytotoxic T cell
CTL: Cytotoxic T cell
G-CSF: Granulocyte-colony stimulating factor
PBMC: Peripheral blood mononuclear cells
PBSC: Peripheral blood stem cel
ST: Streptamer
Th: Helper T cell
