# Supplementary figures and images for: Assessment of the effector function of CMV-specific CTLs isolated using MHC-multimers from granulocyte-colony stimulating factor mobilized peripheral blood
Source: J Transl Med. 2015 May 20;13:165. doi: 10.1186/s12967-015-0515-z (PMC4458005; doi:10.1186/s12967-015-0515-z)

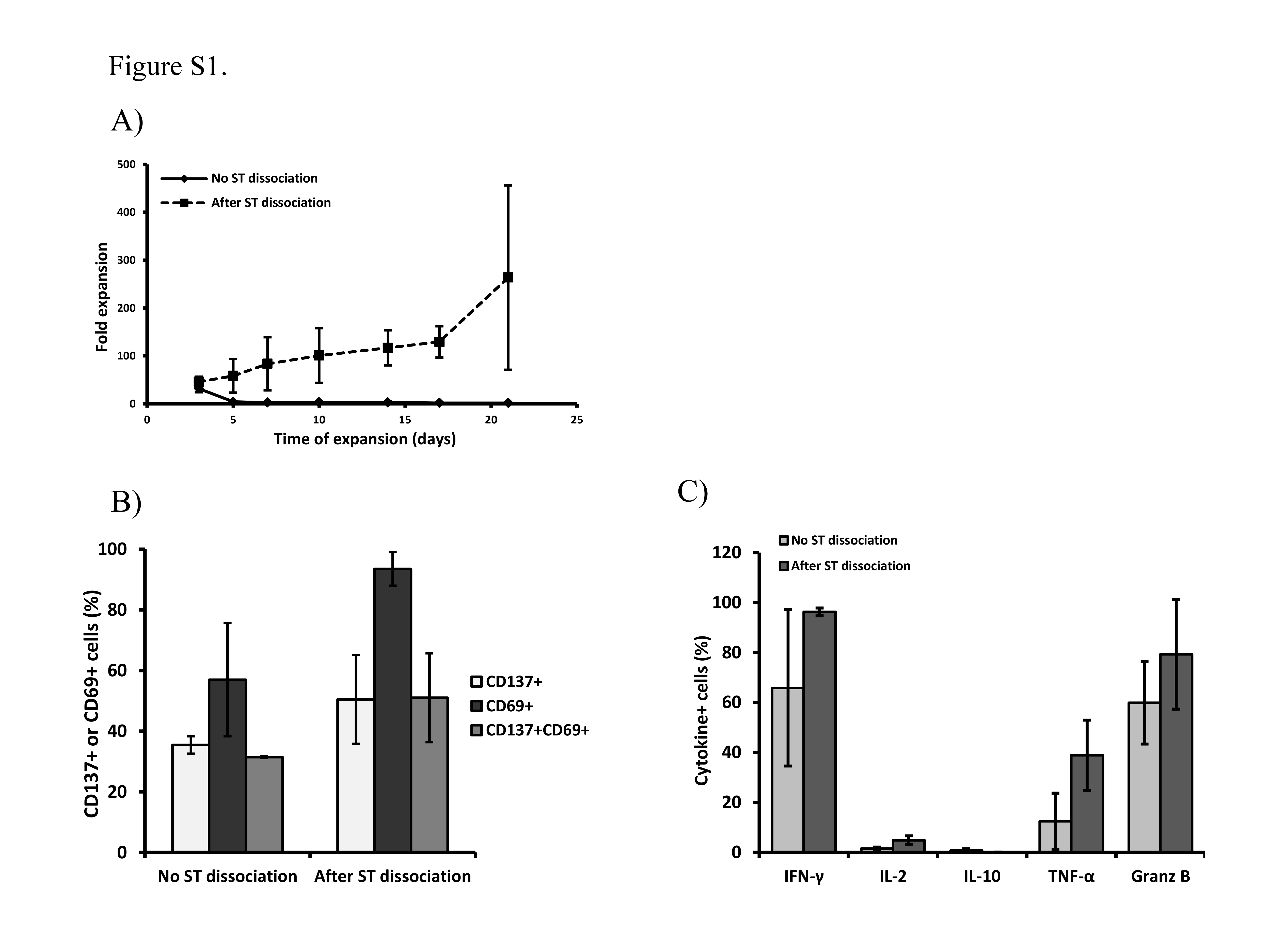

Supplement: Additional file 1: Figure S1. — Expansion and functionality of CMV-CTL with or without ST dissociation. ST was dissociated (n = 3) or left untouched (n = 3) from CMV-CTL isolated from non-mobilized samples, and cells were expanded and functionally characterized afterwards. (A) Mean fold expansion of CMV-CTL obtained from non-mobilized samples, with and without ST dissociation using D-biotin. Activation marker expression (B) and cytokine production (C) upon antigenic re-stimulation in expanded CMV-CTL with ST bound to the cell surface or ST dissociated from the TCR. [file 12967_2015_515_MOESM1_ESM.tiff]
